# FLIM intensity-based image segmentation reveals upregulated energy metabolism and chemotherapy sensitivity in MCF-7 cells

**DOI:** 10.1242/jcs.263702

**Published:** 2025-12-08

**Authors:** Yu-Kai Huang, Mary Zhuang, Michelle A. Digman

**Affiliations:** ^1^ Laboratory for Fluorescence Dynamics, Irvine, CA 92697, USA; ^2^ Department of Biomedical Engineering, University of California, Irvine, CA 92697, USA; ^3^ Department of Biological Science, University of California, Irvine, CA 92697, USA

**Keywords:** Cancer, Mitochondria, Fluorescence lifetime, Metabolism

## Abstract

Mitochondrial transfer to recipient cells triggers a respiratory burst by increasing ATP production and cellular energy metabolism. However, its impact on intracellular metabolic shifts remains unclear. This study introduces a novel methodological approach and new biological insights into mitochondrial dynamics in cancer cells. We developed fluorescence-lifetime imaging microscopy (FLIM) intensity-based image segmentation (FIBIS), an algorithm optimized for single-mitochondrion analysis. FIBIS utilizes NADH autofluorescence, eliminating the need for biomarker staining, and improves mitochondrial detection accuracy by 35% compared to raw intensity thresholding. This method is particularly effective for analyzing dynamic mitochondria in live cells. Using FIBIS, we show that normal epithelial mitochondria uptake alters the free NADH-to-bound NADH ratio, increasing bound NADH in both estrogen- and progesterone receptor-positive and triple-negative breast cancer cells. Additionally, mitochondrial transfer enhances cancer cell sensitivity to oxidative stress-inducing anti-cancer drugs, suggesting a potential restoration of normal reactive oxygen species tolerance. Overall, FIBIS is a robust methodological approach that uses the phasor-FLIM technique to analyze NADH levels (free and bound) at the single-mitochondrion level, providing new biological insights into transferred mitochondrial dynamics in cancer cells.

## Teaser

**Summary:** FLIM intensity-based image segmentation (FIBIS) enables motion-independent single mitochondrion detection in live cells and quantifies the metabolic activity of mitochondria transferred from donor cells.

## INTRODUCTION

Mitochondria play a crucial role in various cellular processes, such as cell growth, proliferation, energy metabolism, reactive oxygen species (ROS) regulation and survival (Green and Reed, 1998; Federico et al., 2012; Jeong and Seol, 2008). In cancer cells, these dysfunctional organelles undergo metabolic alterations, causing cells to shift from oxidative phosphorylation (OXPHOS) to glycolysis, commonly referred to as the Warburg effect. This metabolic adaptation is necessary to meet the high demands for proliferation and to survive in the acidic microenvironments of metastasis (Liberti and Locasale, 2016).

Mitochondrial transfer from normal cells to cancer cells can rescue dysfunctional aerobic respiration, affecting cancer cell behavior. However, the precise mechanisms remain unclear. Current methods for studying this phenomenon have limitations – co-culture models have low transfer rates, isolated mitochondria techniques lack single-organelle resolution, traditional fluorescence microscopy requires potentially disruptive labels, and electron microscopy (EM) cannot capture real-time dynamics in live cells. These constraints highlight the need for advanced techniques to elucidate the effects of mitochondrial transfer on cancer cell metabolism at a single-organelle level in live cells. This study has two primary objectives: (1) to develop and validate a novel fluorescence-lifetime imaging microscopy (FLIM)-based methodology for analyzing single-mitochondrion metabolism, and (2) to apply this method to investigate the biological effects of mitochondrial transfer on breast cancer cell (BCC) metabolism and drug sensitivity.

It is known that dysfunction of aerobic cellular respiration can be rescued by the transfer of mitochondria donated from normal cells (Kim et al., 2018; Li et al., 2017; Newell et al., 2018; Spees et al., 2005; Wang and Gerdes, 2015), and that this enables the adaptation of cancer cells within the tumor microenvironment by increasing their proliferative capacity, drug resistance and invasiveness (Caicedo et al., 2015; Chang et al., 2019; Hekmatshoar et al., 2018; Pasquier et al., 2013; Zampieri et al., 2021). However, reports show that BCCs with epithelial cell-donated mitochondria, as opposed to other dontated mitochondria, have suppressed proliferation, are less invasive and have an increase in drug sensitivity (Elliott et al., 2012; Ivers et al., 2014; Spink et al., 2006). In the seminal study conducted by Spink et al., it was revealed that estrogen-responsive BCCs, when subjected to co-culture with epithelial cells, engage in a competitive interaction for limited substratum resources, particularly with respect to epidermal growth factor (EGF). The consequential stimulation of epithelial cell proliferation was attributed to the release of transforming growth factor-α (TGF-α) from estrogen-responsive BCCs, thereby suppressing the growth of BCCs. Subsequent investigations by Ivers et al. provided pivotal video evidence, depicting epithelial cells enveloping BCC aggregates, and electron microscope (EM) images that highlighted the abundance of mitochondria within BCCs co-cultured with epithelial cells. This work underscored the potential that mitochondria transfer to host cells can prompt such cells to transition to a differentiated state and cause subsequent loss of malignancy as the expression of the monocarboxylate transporter-1 (MCT1, also known as SLC16A1), a membrane protein which transports lactate and pyruvate, was observed to diminish.

Notwithstanding the informative insights gained from co-culture models, a significant limitation of co-culture condition lies in the notably low and unpredictable rate of mitochondria transfer. To address this challenge, recent scientific advancements have popularized the utilization of mitochondria isolation as an alternative method for investigating host cell behavior and metabolic alterations following the transplantation of mitochondria. In a noteworthy contribution to this line of research, Chang et al. (2019) employed the mitochondria isolation approach, unveiling promising findings wherein BCCs with transplanted mitochondria exhibit heightened drug sensitivity, increased oxygen consumption rates (OCRs) and enhanced antioxidant enzyme activity. Although analysis of mitochondria transfer is well established, the mechanism through which adapted epithelial mitochondria drive BCCs back to normal metabolic function remains poorly understood due to limited one-dimensional data for each measurement (Zampieri et al., 2021). For example, the Agilent Seahorse ATP real-time rate assay measures the different mitochondria complex respiratory rate of the whole-cell culture using inhibitors across time but lacks the ability to verify viability and function of individual transferred mitochondria after entering recipient cells. EM enables the high-resolution imaging of mitochondria but lacks the ability to visualize mitochondria morphology in real time. To quantify successful consumption of exogenous mitochondria, fluorescence microscopy is commonly used to analyze time-lapse videos of pre-labeled mitochondria (Ivers et al., 2014). Although biological analysis requires labeling of the mitochondria with multiple fluorescent probes for specific mitochondrial functions, researchers have not fully realized the potential of using morphological changes in individual mitochondria to suggest metabolic activities, thereby allowing their dynamics to be differentiated depending on the extent of being absorbed by host cells (Caicedo et al., 2017; Torralba et al., 2016; Zampieri et al., 2021). Notably, analysis of transferred mitochondria using exogenous dyes, such as tetramethylrhodamine methyl ester (TMRM), to measure the inner and outer membrane potential, or CellROX, which detects superoxide to imply reactive oxygen species (ROS) level, does not provide a comprehensive physiological native change to all mitochondria, as that requires a concentration that has to be optimized for each cell type. As an alternative, we have used the intrinsic autofluorescence co-factor, nicotinamide adenine dinucleotide (NADH) lifetimes to determine the free versus protein-bound ratio of this substrate. The advantage of NADH is that the structure of the free and bound form changes from a short to long fluorescence lifetime, respectively. Bird and co-workers (2005) were the first to notice a significant relationship between the lifetime ratio and the NADH-to-NAD^+^ redox ratio within BCCs. This is known as the ‘metabolic trajectory’, a term coined by Stringari et al. who demonstrated that the quantification of the form of NADH indicates cellular energy metabolism using the fit-free phasor approach to FLIM (Stringari et al., 2011).

To capture mitochondria metabolic activity and morphological changes simultaneously in real-time, we developed a FLIM intensity-based image segmentation (FIBIS) algorithm to mask out individual mitochondria from NADH FLIM intensity images. This requires optimization of intensity filtering parameters and object recognition threshold based on object size. We demonstrate that single mitochondria can be isolated from large intensity blur of cytosolic NADH, caused by movements when acquiring FLIM images, and provide the metabolic index with the integration of phasor image arrays.

**Fig. 1.**
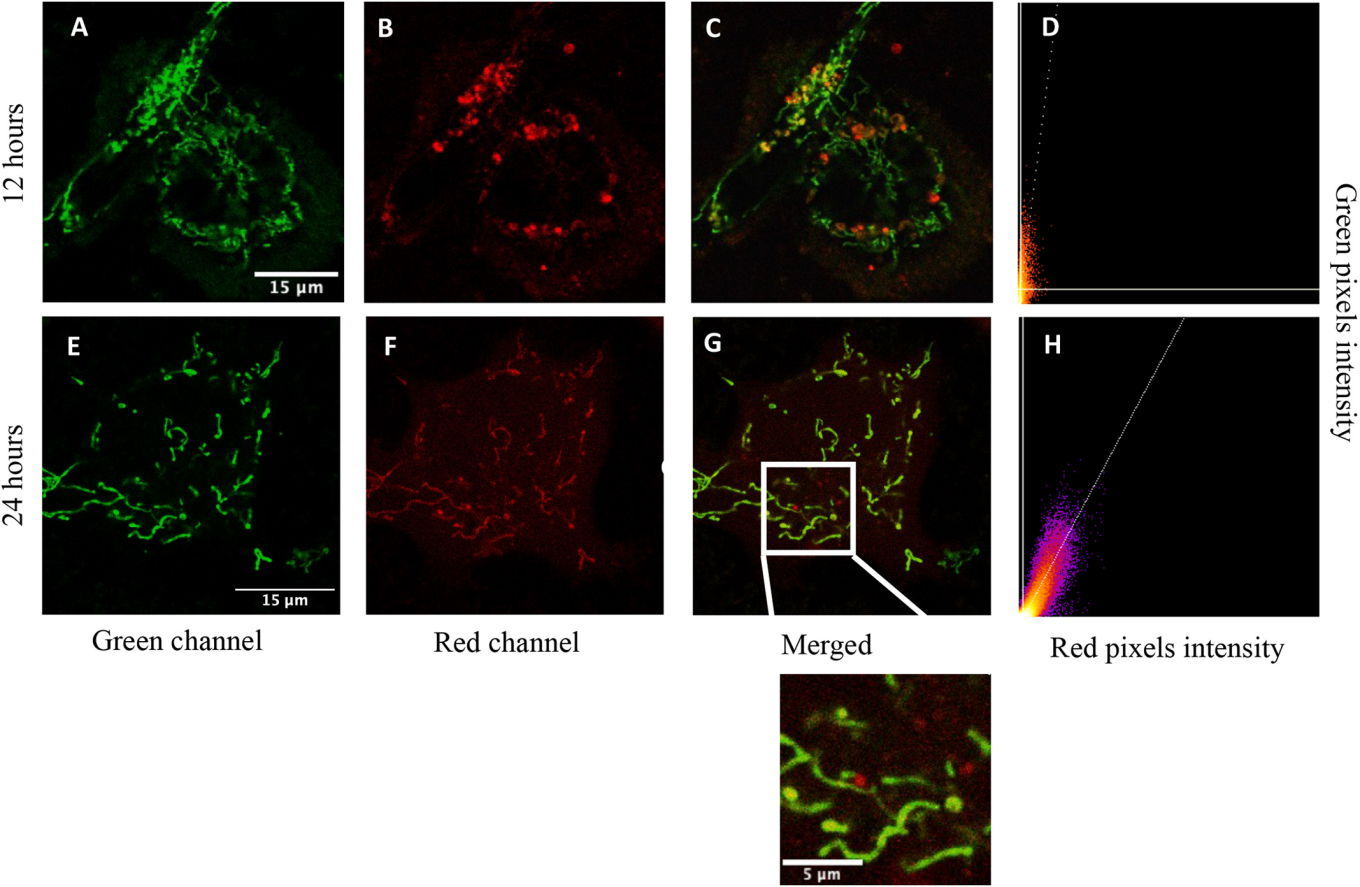
**Fluorescence images of mitochondria transferred to BCC.** Fluorescence images of COX8–GFP-transfected BCCs co-cultured with isolated Mito7–mRuby-labeled mitochondria for 12 h and 24 h, respectively. (A) Endogenous mitochondria transfected with COX8–GFP represented in green channel and (B) exogenous mitochondria labeled with Mito7–mRuby represented in red channel. (C) Merged channels differentiate the two signals, which is quantified using colocalization scatter plots after 12 h of incubation (D). (E–H) As for A–D but after 24 h of incubation time, exhibiting the fusion of two expressions. Merged images present Pearson's R of 0.59 and 0.72 in post 12 h and 24 h incubation respectively. Costes randomization value: 10, *P*-value=1 (significant of colocalization >0.95). Images representative of ten images per biological replicate (*N*=3).

To verify that the NADH FLIM phasor shift represents changes of metabolism, we inhibited the OXPHOS and glycolysis pathways in BCCs and measured the NADH lifetime shifts on the phasor plot. Results showed that treatment with the glycolytic inhibitor, 2-deoxyglucopse (2DG), increased the bound fraction of NADH in BCCs with epithelial transferred mitochondria. When we treated the BCC cells with the OXPHOS inhibitor, rotenone, the fractional contribution of free NADH increased signifying a shift towards glycolysis. The OCR of MCF7 cells with transferred mitochondria increased and, at the same time, these cells showed a decrease in extracellular acidification rate (ECAR). We observed that after mitochondria have been transferred into host cells, the transfected labeling signal from genes of donor and host cells fused together (as shown in Fig. 1) and hence there is a new mitochondria ‘product’, establishing even more capacity to energy production. The phasor approach FLIM links both spatial resolution and metabolic index in one system. Additionally, this work enables specific targeting of the mitochondrial metabolic state with our FIBIS algorithm.

## RESULTS

### Artificially isolated mitochondria taken up by breast cancer cells increases drug sensitivity

Rustom et al. were the first to find out that organelles, including mitochondria, can be transported through tunneling nano-tubules (TNTs) (Rustom et al., 2004). However, the number of transfer events is small and hence the probability of being able to monitor an event is low, and it is difficult to visualize events in real time. An understanding of the effects and impact of individual transferred mitochondria with respect to the energy metabolism of single host cells has not yet been well established. To monitor these events, we applied an artificial mitochondria isolation process, through chemical gradient centrifugation, to obtain pure mitochondria from normal breast epithelial cells. We proved that artificially isolated mitochondria entered BCCs as shown in Fig. S1, as epithelial mitochondria labeled with COX8–GFP were present in stable mCherry-expressing MDA-MB-231 cell cultures. Furthermore, we expected host cells that consumed mitochondria would show a homogenous fused mitochondria product, given that membrane proteins diffuse in the order of <0.1 μm^2^/s (Appelhans and Busch, 2017). Hence, we labeled epithelial and BCC mitochondria with Mito7–mRuby and COX8–GFP, respectively, using temporal transfection (Fig. 1A,B, 12 h incubation; Fig. 1E,F, 24 h incubation) to prevent dye transfer or diffusion across time, which occurs with MitoTrackers. As expected, the transferred mitochondria fused with the host mitochondria in a time-dependent manner (Fig. 1C,G), creating an apparent homogenously distributed green and red protein population, which was observed with our diffraction limited confocal imaging system (Fig. 1G). COX8–GFP and Mito7–mRuby colocalization was measured with an average Pearson's R of 0.593 (±0.155, s.d.) and 0.686 (±0.114, s.d.) at 12 and 24 h, respectively, using ImageJ indicating a moderate positive colocalization (Fig. 1D,H). The results showing fused new products provide proof on how transferred mitochondria blend into host cells and manage to eventually change BCC metabolism.

**Fig. 2.**
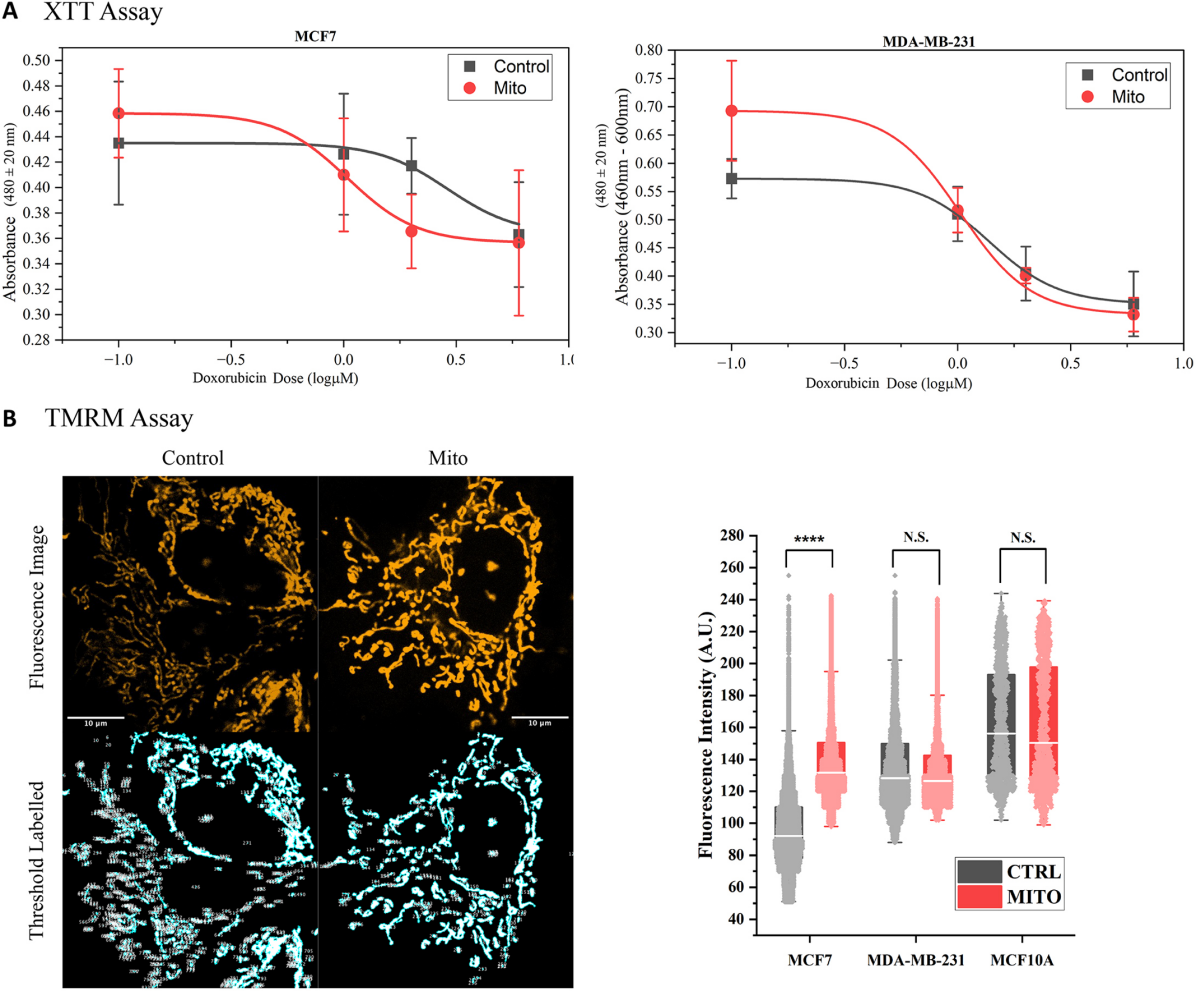
**Cell viability and mitochondrial membrane potential analysis after 3 days of incubation.** (A) Cell viability test for BCCs treated with 0, 1, 2 and 5 μM of doxorubicin after 3 days as performed with a XTT assay. IC50 values of MCF7 control and mitochondria (Mito) groups were 2.30 μM and 1.10 μM. MDA-MB-231 control and mitochondria groups were 1.48 μM and 0.95 μM. Results are mean±s.d. (*N*=4). (B) Single mitochondrial TMRM intensity analysis. A significant increase was found for the MCF7 mitochondria group indicating enhanced mitochondrial activity. *N*=9211, 7907, 4185, 5670, 5350, and 6124 for MCF7 control mitochondria, MDA-MB-231 control mitochondria, MCF10A control, and MCF10A mitochondria respectively. Data are represented as mean and interquartile ranges (highlighted by the shaded box). Whiskers show 5–95th percentiles. A.U., arbitrary units. ****P<0.00001; N.S., not significance (two sample Kolmogorov–Smirnov test).

On the perspective of isolated mitochondria, we tested whether mitochondria were still active when isolated from donor cells into the cell culture medium, given that they are immediately transported into BCC cultures. We targeted successful transferred mitochondria host cells as mentioned in Fig. S1 and measured the TMRM intensity to compare with controls. Transferred mitochondria were verified to remain active after entering host cells by staining with TMRM (Fig. 2A). Interestingly, MCF7 cells were found to have a significant increase in TMRM intensity levels after mitochondria transfer, suggesting enhancement of proton pumps in complexes I and III, which are essential components for OXPHOS (Zorova et al., 2017). MCF10A and MDA-MB-231 cells with added epithelial mitochondria did not present significant changes suggesting that their respiration rates were still at normal levels. Further investigation was done through quantifying the change of mitochondrial metabolic state influenced by exogenous mitochondria. To control for the amount of mitochondria being isolated and co-cultured with BCCs, we performed a Bradford assay confirming that the amount of isolated mitochondria was consistent at ∼50–200 μg/ml (protein concentration, Fig. S1C), which is within the range of typical mitochondria transfer experiments (Caicedo et al., 2015). However, not all BCCs fully uptake transferred mitochondria even in the absence of nutrient-depleted medium, which brings in the need of fluorescence microscopy to verify mitochondria uptake by BCCs. The number of individual isolated mitochondria located outside the host cells exhibited a decrease over time, indicating consumption within the cell culture (Fig. S1D). Movie 1 exhibits how a BCC host cell with transferred mitochondria continues to consume adjacent isolated mitochondria expressing GFP. To prevent the possibility of cross-transfection of protein plasmids into BCCs after artificial mitochondria isolation, paxillin–GFP was labeled to ensure the proper purification of mitochondria. The tile images presented a wide area of the BCC culture, where host cells were stained with MitoTracker Deep Red, and a minimal amount of paxillin–GFP was observed (Fig. S1E). This observation suggests that although the isolated mitochondria might contain a small number of other substances, the transfection of protein plasmids between epithelial cells and BCCs is unlikely to occur.

We discovered that completely consumed mitochondria exhibit an elongated morphology instead of a punctate round shape, implying that the uptaken exogenous mitochondria display a healthy or active phenotype at post 24 h of incubation. Interestingly, TNT-like structures were found only in the mitochondria-transferred condition (Fig. S1F,G) where they transported the pre-labeled epithelial mitochondria; this suggests that the consumption of epithelial mitochondria not only occurs through endocytosis of isolated mitochondria but also cell–cell interaction.

To investigate the impact of epithelial mitochondria on BCC cell death, a cell viability assay was performed on BCCs treated with the anti-cancer drug, doxorubicin, at various concentrations (Fig. 2B). IC50 values for MCF7 cells dropped from 2.30 μM to 1.10 μM after mitochondria transfer and those for MDA-MB-231 dropped from 1.48 μM to 0.95 μM. The results of the assay revealed that the cell proliferation of BCCs containing epithelial mitochondria was enhanced, as we observed a higher absorbance in raw data, but concurrently, these cells became more sensitive to drug treatments. This outcome was anticipated given the observed fusion of transferred mitochondria with endogenous mitochondria, thereby re-establishing normal mitochondrial antioxidant defensive mechanisms and consequently losing tolerance to high levels of oxidative stress (Fig. S2). Moreover, the combination of transferred mitochondria and doxorubicin resulted in more cell death than seen with metabolic inhibitors alone (Fig. S2). This discovery highlights the potential of mitochondria transfer in therapy, as it enhances sensitivity to anti-cancer drugs.

### FLIM intensity-based image segmentation

**Fig. 3.**
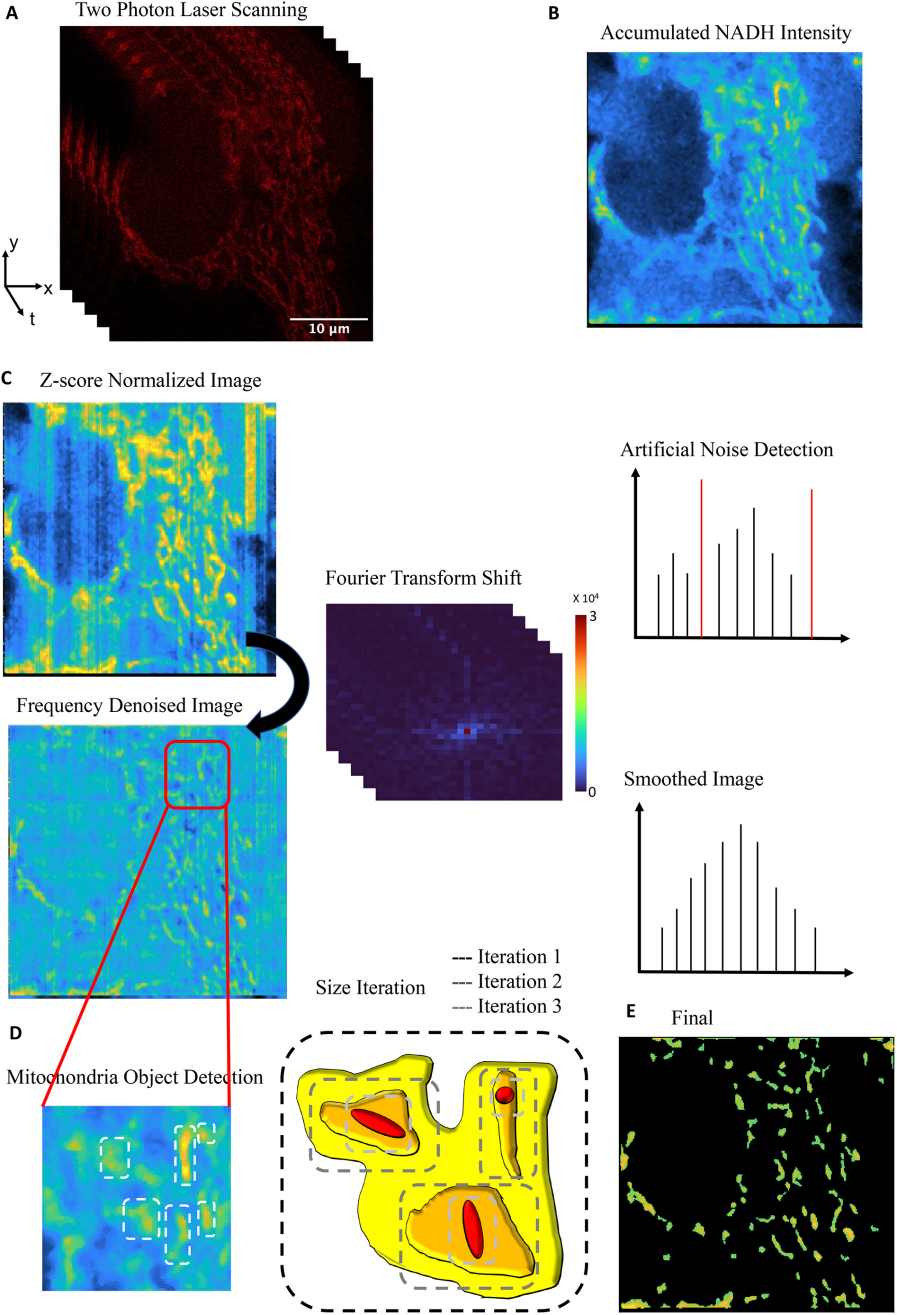
**Flow chart of FIBIS.** (A) Experimental images are line scanned by two-photon laser excitation for 15 frames in total to collect sufficient photon arrival time resulting in (B) the accumulated intensity image. (C) Highlights pixels from images as in B that are z-score normalized, which amplifies artificial laser scanning noise. FIBIS detects artificial noise through assessing abnormal histogram peaks and determines optimal frequency smoothing from a Fourier transform shift to set up a threshold level. (D) Objects that are larger than a predefined size go through size iteration following intensity gradient, which results in (E) the final masked image.

**Fig. 4.**
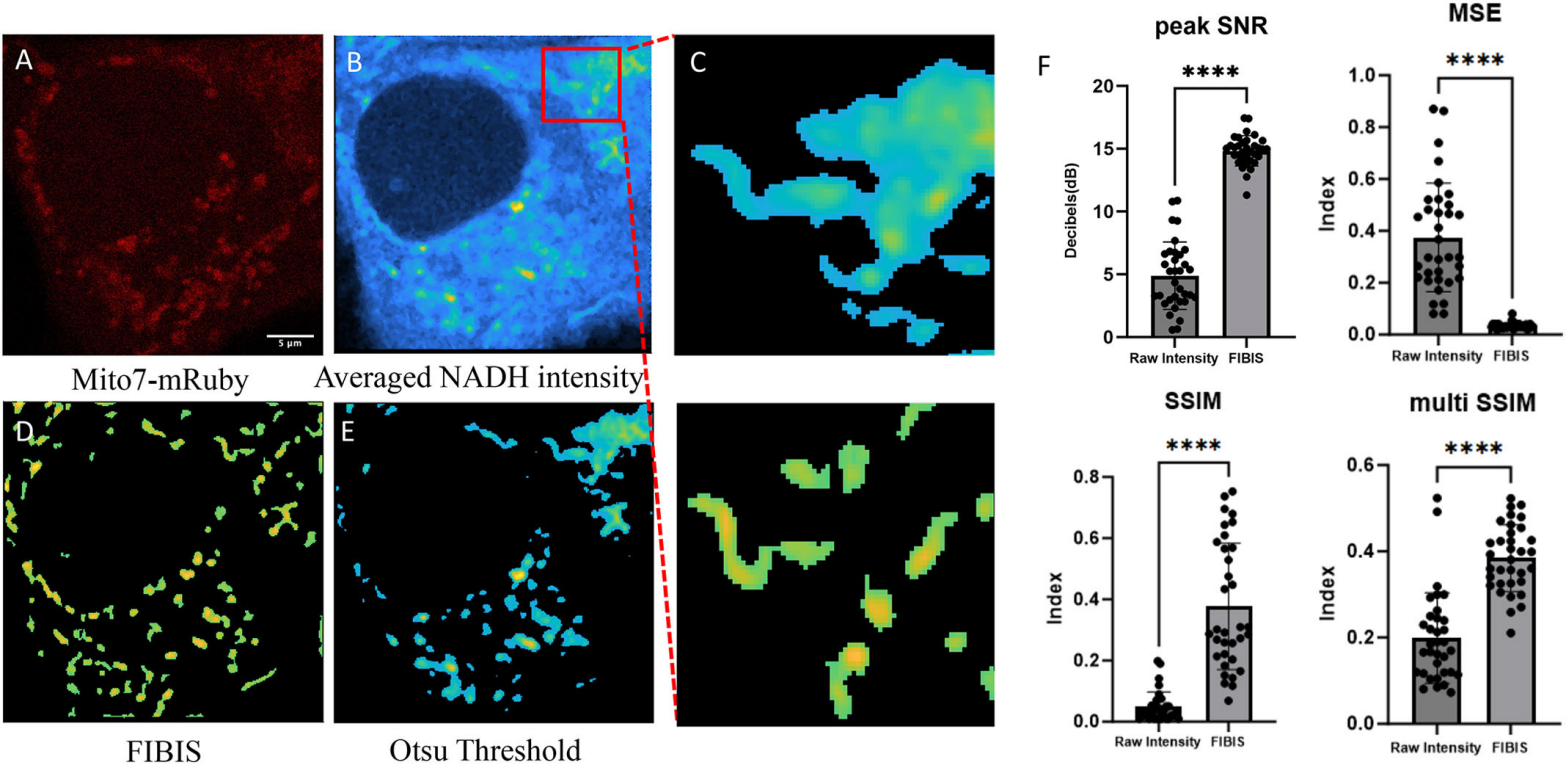
**FIBIS segmentation of NADH auto-fluorescence intensity.** (A) Fluorescence confocal image and (B) FLIM NADH intensity image of transferred mitochondria consumed by a MCF7 recipient cell (in mRuby channel) post 24 h. (C) Large blobs were iteratively removed using image erosion with a cutoff greater than the measured mitochondrial size, leading to the final segmentation (bottom). (D) Final image of NADH intensity-based image segmentation. (E) Otsu threshold after normalization at the 0.9 quantile where large blobs were not able to resolve based on intensity threshold. Scale bar: 5 μm. (F) Image similarity test performed by referencing the fluorescence confocal image. Results are given as mean±s.e.m. (*n*=34). ****P<0.0001 (two-tailed paired sample t-test).

The phasor approach to FLIM was applied to quantify the shift of mitochondrial metabolic state through calculating the linear fraction of free to bound NADH. Although previous works have demonstrated the quantification of single-cell level metabolic index (Mah et al., 2018; Ranjit et al., 2019; Trinh et al., 2017), this work focus only on single mitochondria. To target only the mitochondria NADH phasors, we developed a FLIM intensity-based image segmentation (FIBIS) method. To acquire sufficient photon counts to make a histogram of lifetime decay, confocal microscope scans across ROI frames are performed 20 times, which creates an accumulated NADH intensity along with modulation and phase arrays saved in R64 files (Fig. 3A,B). These are used for later transformation to phasor analysis. However, the accumulated NADH intensity leads to two major problems when dealing with mitochondrial dynamics. First, fast-moving mitochondria will have traveled across ROI, and the tracks create a motion blur in the final accumulated NADH intensity. Second, during multiple scanning of the image, relatively high intensity pixels broaden the intensity range of the image making it difficult to determine the optimized cutoff for image thresholding. To mitigate the diminishing problem, we applied range normalization to highlight all NADH intensity signatures across scanned frames. However, this will unavoidably amplify artificial noise across the multiple scanned frames (Fig. 3C). To smooth discontinuous vertical noise and not lose highlighted signatures, frequency peaks were picked out of Fourier-transformed (FFT) noisy images (Wahab et al., 2021; Wang and Liu, 2010) to produce a normally distributed histogram (Fig. S3). Pixels within the interquartile intensity range were selected, and Otsu's method was then applied to determine the threshold and subtract out diffusion background. Furthermore, mitochondria size was set at a reasonable range (0.5–7 μm) to eliminate noise pixels or activate erosion iteration for objects with high motion blur (Fig. 3D), with the final production serving as a mask for FIBIS segmentation (Fig. 3E). A detailed flowchart of the FIBIS image processing is shown in Fig. S3. To quantify optimization of FIBIS method, raw intensity and FIBIS images were compared with their corresponding Mito7–mRuby fluorescent-labeled images as reference using peak signal-to-noise-ratio (peak SNR), mean square error (MSE), a structural similarity index measure (SSIM) (Wang et al., 2004) and multi SSIM parameters. MSE measures the absolute pixel-wise error between the two images to quantify intensity shifts and background artifacts (Eqn 1), where raw intensity exhibits a significant larger error than FIBIS. Peak SNR is particularly sensitive to minor pixel variations, such as artificial noise, as described by Eqn 2. A higher PSNR value was measured in FIBIS-processed images than in averaged NADH images, indicating greater similarity between the reference, signifying lower distortion. Finally, SSIM is applied to compare the structural similarity between processed and reference images, providing a quantitative measure of image quality. Multi-SSIM further detects blurriness in images caused by mitochondrial dynamics, where FIBIS effectively resolves these issues without introducing distortion. It is notable that the emission spectrum of NADH and mRuby were widely separated to prevent signal overlapping (Fig. S4A). FIBIS performed a better structure comparison than raw intensity overall in the 34 trials (Fig. 4) bringing a 35% increase in average SSIM and 18% in average multi SSIM. In short, the FIBIS method helps phasor FLIM users to robustly crop and label individual mitochondria with their fraction index on the free-to-bound NADH trajectory.
(1)

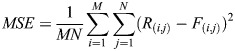

(2)

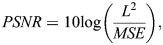
where M,N are the image dimensions. *R*_(*i*,*j*)_ and *F*_(*i*,*j*)_ are pixel intensity values of reference and sample images. *L* is the maximum pixel intensity value from a 0–255 range.

### Increase in the bound NADH fraction insinuates dependence on OXPHOS metabolism in mitochondrial enhanced MCF7 cells

**Fig. 5.**
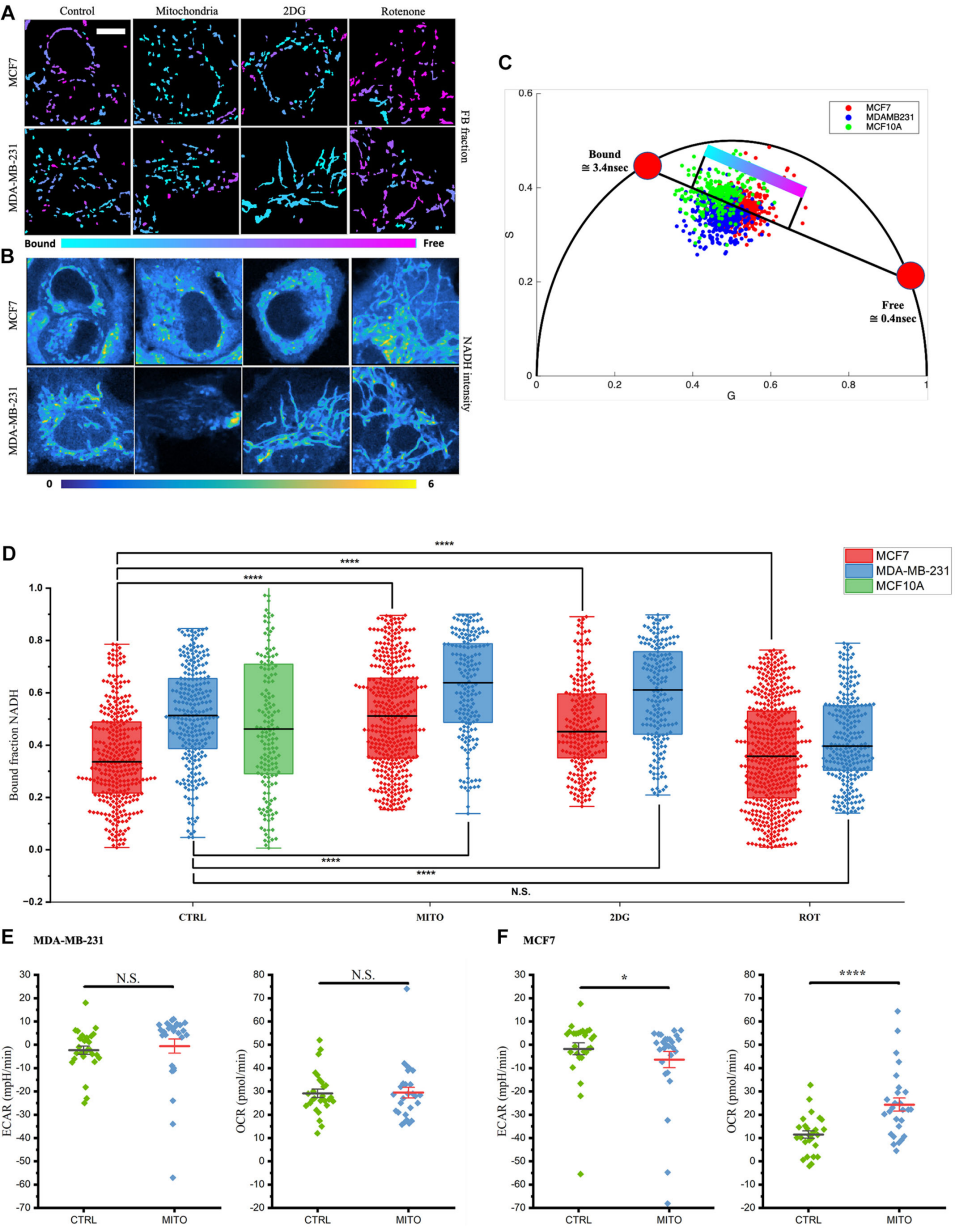
**Phasor FIBIS metabolic index and Seahorse assay analysis.** (A,B) MCF7 and MDA-MB-231 cells were subjected to four different conditions: DMSO, transferred mitochondria, 2DG and Rotenone. (A) Segmented mitochondria overlaid with pseudocolored referenced from the phasor plot for the free and bound fraction of NADH. (B) Raw NADH intensity images of BCCs. Scale bar: 10 μm. Shown are selected images from four repeats. (C) Phasor plot of MCF7, MDA-MB-231 and MCF10A control from experiments as in A and B. Red points represent free to bound NADH trajectory where extreme points are set at 0.4 and 3.4 ns respectively. Data points outside of eclipse distribution are counted as outliers and are not included in free and bound fraction NADH analysis. (D) Scatter plot with overlaid boxplot of the segmented mitochondria free and bound (FB) fraction. Only positively recognized mitochondria were counted as data points in the transferred mitochondria group. *n*=612, 490, 538, and 589 distinct mitochondria in MDA-MB-231 control, transferred mitochondria, and 2-deoxyglucose and rotenone treatment groups, respectively. *n*=915, 765, 723, and 872 distinct mitochondria in MCF7 control, transferred mitochondria, and 2-deoxyglucose and rotenone treatment groups, respectively. *n*=877 in MCF10A control. The box represents the 25–75th percentiles, and the median is indicated. The whiskers show the complete range. N.S., not significant; ****P<0.00001 (two sample Kolmogorov–Smirnov test). (E,F) Oxygen consumption rate and Extracellular acidification rate (ECAR) and of normal and transferred mitochondria. *P*-values are calculated using two-tail paired Student's *t*-test. *N*=45. * indicates *P*<0.05 and **** indicates *P*<0.00001. Data are represented as mean±s.d. ranges.

FIBIS masked images were used to generate mitochondrial properties including the sample group, size and the metabolic phasor coordinates (s,g). The lifetime of NADH with a concentration of 2.5 μM was measured to calibrate the pure population of free NADH in 10 mM NaH_2_PO_4_ solution, Fig. S4B shows the single exponential lifetime at 0.4 ns and ∼3.5 ns with pure mitochondria indicating bound NADH on the phasor plot (Ma et al., 2016). The free and bound fraction of NADH reveals a 42% increase in the fractional contribution of bound-state NADH for MCF7 cells and 20% for MDA-MB-231 cells at 24 h after mitochondria transfer (Fig. 5D). The increased bound NADH fraction is identical to that seen with BCCs treated with the glycolytic inhibitor, 2-deoxyglucose (Cheng et al., 2012). This suggests that mitochondrial enhancement changes cellular respiration towards the OXPHOS state. By contrast, the fraction of bound NADH decreases when treated with OXPHOS inhibitor, Rotenone, which shows the ability of cancer cells to switch between metabolic states freely to meet self-growth. To verify that the NADH FLIM signature does indeed suggest that there is metabolic shift, we compared the results with gold standard metabolic assay measurements. We analyzed the OCR and ECAR using the Seahorse XF analyzer. MCF7 cells with additional transferred mitochondria showed a significant increase in OCR and decrease in ECAR, implying the shift of metabolism towards OXPHOS (Fig. 5E,F). However, MDA-MB-231 cells did not show significant changes in the Seahorse XF Analyzer, which could be explained by their aggressive phenotype. Triple-negative breast cancer (TNBC) leads to relatively higher respiration to support their higher demand on proliferation and migration (Sun et al., 2020). This was shown here as MDA-MB-231 cells had relatively higher values for both OCR measurements (Fig. 5F) and the bound fraction of NADH (Fig. 5D) compared to that in MCF7s. This finding also suggests that the Warburg effect does not consequently mean that cancer cells fully rely on glycolysis for their energy metabolism but still maintain OXPHOS metabolism, which is activated according to cancer phenotypes. Notably, the FIBIS segments individual mitochondria from the NADH intensity image (Fig. 5B) and pseudocolor masked objects (Fig. 5A) by projecting a color bar on the trajectory line of the phasor plot (Fig. 5C). This could aid the investigation of separating local and transferred mitochondria in the host cell.

Given the free and bound fraction of NADH in mitochondria, we tried to use FIBIS to distinguish between endogenous and exogenous mitochondria. When assessing the fluorescence images of transferred and local mitochondria mentioned in the previous section, there were no significant difference or threshold for the free and bound fraction that could be set to distinguish between the two sources (Fig. 5A) in fully consumed host cells. This implies that exogenous mitochondria have gone through numerous fusion events after entering BCCs (Li et al., 2017; Ni et al., 2015), hence inheriting the functional electron transport chain complexes bringing back the bound-state fraction of NADH. In short, the free and bound fraction seen in FIBIS distinguishes the metabolic change of individual mitochondria from NADH autofluorescence lifetime, an improvement in resolution of the phasor approach to FLIM.

## DISCUSSION

The rise in research of mitochondria transfer has led the biomedical field to explore therapeutic approaches towards various diseases (Liu et al., 2022). Despite the well-established approaches to quantify isolated mitochondria amount or quality, it remains unclear how isolated mitochondria affect the host cell at the single-cell scale. Studies have shown that the majority of stem cells upregulate their metabolism, which in turn, can facilitate cancer cell growth (Caicedo et al., 2015; Newell et al., 2018; Wang and Gerdes, 2015). Conversely, research into epithelial cells indicates that their mitochondrial transfer can enhance drug sensitivity in cancer cells, leading to a heightened response to chemotherapy (Chang et al., 2019). Additionally, another study has found that breast cancer cells adopting mitochondria from epithelial cells exhibited a less malignant phenotype (Ivers et al., 2014). Therefore, the question arises of does the upregulated metabolism in cells with chemotherapy sensitivity contribute to their increased responsiveness to these drugs. Understanding this relationship could be crucial for developing targeted treatments in breast cancer therapy.

To obtain a clear visualization and segmentation of individual mitochondria we developed FIBIS, an intensity-based segmentation method, coupled with the phasor approach to NADH FLIM and target only mitochondrial metabolism in live cells. FIBIS detects the artificial frequency noise, which stems from the accumulation of frames when acquiring FLIM data and eliminates the intensity of blurred pixels caused by mitochondrial movement. In addition, FIBIS achieves robust recognition of single mitochondrial objects for further free and bound fraction analysis. The final product is an image of single mitochondria which is used as a mask to phasor coordinate arrays enabling the calculation of the free and bound NADH fraction. In our findings, BCCs gained more bound fraction of NADH after transfer of epithelial mitochondria, which suggests that they tend to switch their metabolism more to the OXPHOS state. Initially, we assumed that free and bound fraction of NADH could enable us to distinguish between endogenous and exogenous mitochondria in the host cell. However, FLIM images did not show a clear difference between the two sources. This is expected to be caused by the fusion of exogenous and endogenous mitochondria facilitated by Mfn1, Mfn2 and optic atrophy 1 (OPA) (Cowan et al., 2017), which results in the overlayed channel of colors in mitochondria shown in Fig. 1G. By that, fused mitochondria inherited functioning mitochondrial complex results in the increase in the bound fraction for NADH. Free and bound fraction results were also confirmed through the Seahorse XF Analyzer where MCF7 cells showed a significant increase in OCR. Interestingly, MDA-MB-231 cells did not present any significant trend in proton flux, which could be due to their aggressive phenotype. This has been mentioned in other literature (Evans et al., 2021; Saha et al., 2021; Sun et al., 2020), which implies that cancer cells do not all follow the Warburg effect to sustain energy but switch between metabolic states accordingly to meet their needs. Although BCCs shift to a more OXPHOS metabolism after uptaking epithelial mitochondria, cell viability decreased as compared with what was seen with other inhibitors and anti-cancer drugs, which makes epithelial mitochondria a great interest source for therapeutic applications.

The introduction of normal epithelial mitochondria might indeed help restore a more normalized metabolic state in cancer cells. This ‘normalization process’ might happen because during the fusion of exogenous and endogenous mitochondria, the outer membranes combine, likely incorporating functional OXPHOS complexes from the exogenous mitochondria into the final fused organelle. This process might effectively transfer and integrate healthy respiratory chain components into the cancer cell mitochondria. These mechanisms together could lead to an enrichment of healthy, functional mitochondria including selective mitophagy (Twig and Shirihai, 2011) within the cancer cells, thereby enhancing their overall OXPHOS capacity (Dong et al., 2023; Sansone et al., 2017).

The development of this work provides a spatial mapping of single mitochondria metabolic state to investigate transferred mitochondrial metabolism dynamics. The convenience of the phasor approach FLIM creates a direct visualization of the single mitochondria condition. As it provides the image property, we will make further use of the mitochondrial morphology. Future experiments will combine the use of metabolic index with mitochondrial dynamics analyzed using our MATLAB algorithm, Mitometer (Lefebvre et al., 2021), to correlate fission and fusion dynamics (Peng et al., 2011) with transferred mitochondria samples. Currently, we have analyzed the branching, length and numbers of mitochondrial dynamics compared with fission and fusion activator treatments (Fig. S5). These data suggest that transferred mitochondria are capable of regaining functional selectivity, undergoing mitophagy and engaging in mitochondrial biogenesis, as indicated by a significant increase in mitochondrial number (Fig. S5C). Overall, this work demonstrates the potential to determine metabolic shift of BCCs using microscopy imaging and provide an alternative microscopy method to investigate mitochondria transfer outcome in other varieties of mitochondrial dysfunction disease, such as Alzheimer (Nitzan et al., 2019), neural (Berridge et al., 2016; Gao et al., 2019; Ren et al., 2022), cardiac (Pour et al., 2020) disease and inflammatory (van der Vlist et al., 2022) disease. Combining the TMRM analysis of individual mitochondria and the gold standard Seahorse assay results, we have demonstrated that comparatively FIBIS is a suitable and sensitive method to quantify the mitochondria metabolic state and dynamics at the single mitochondrion level.

## MATERIALS AND METHODS

### Cell culture and artificial mitochondria isolation

MCF10A cells were cultured in Dulbecco's modified Eagle's medium (DMEM/F12) with high glucose, sodium pyruvate and L-glutamine (11320033 Thermo Fisher Scientific, Waltham, MA, USA) supplemented with 5% horse serum (26050088 Thermo Fisher Scientific), 20 ng/ml epidermal growth factor (PHG0313, Thermo Fisher Scientific), 0.5 mg/ml hydrocortisone (H0888 Sigma-Aldrich), 100 ng/ml cholera toxin (9012-63-9 Sigma-Aldrich), 10 μg/ml insulin (I3536 Sigma-Aldrich) and 1% penicillin-streptomycin 100× solution (25-512 Genesee Scientific, San Diego, CA, USA). MCF7 and MDA-MB-231 cells were cultured in DMEM with high glucose, L-glutamate and sodium pyruvate supplemented with 10% heat-inactivated fetal bovine serum (10082147, Thermo Fisher Scientific) and 1% penicillin-streptomycin 100× solution. The mCherry MDA-MB-231 stable cell line was acquired from Dr Olga V. Razorenova (Molecular Biology and Biochemistry, University of California Irvine, USA); all other cell lines were acquired from the ATCC. Cells were incubated at 37°C, 5% CO_2_. Mitochondria were isolated using mitochondria isolation kit for cultured cells (89874, Thermo Fisher Scientific). MCF10As were cultured in T75 flasks for 3 days and acquired 5×10^6^ cells before any labeling or treatment. Centrifugation was done according to the Thermo Fisher Scientific kit protocol in a 4°C temperature cold room and other transportation process were done on ice.

### Bradford assay

Mitochondrial protein concentration was measured by Bradford assay (23236, Thermo Fisher Scientific) to quantify the amount of isolated mitochondria per trial. The standard curve was referenced with bovine serum albumin (BSA, A7906 Sigma-Aldrich) at concentrations of 0, 25, 125, 250 and 500 μg/ml diluted in DPBS (25-508 Genesee, USA). Absorbance at 560 nm was measured on a spectrophotometer Nanodrop 2000 (ND2000CLAPTOP Thermo Fisher Scientific, USA), at least four data points were acquired at each concentration to produce the standard curve.

### Confocal fluorescence imaging acquisition

Mitochondria fluorescence images of MCF10A, MCF7 and MDA-MB-231 cells were acquired on a Zeiss LSM 880 with a 63×, numerical aperture 1.4, oil-immersion objective at frame size of 256×256 and 512×512 px. Sequential scanning was applied to avoid overlapping of excitation between different laser wavelengths. For verification of transferred mitochondria uptaken by BCCs, MCF10A were pre-transfected with COX8–GFP to label epithelial mitochondria and the stable mCherry-expressing MDA-MB-231 cell line was used as targeted recipient cells to detect fully consumed mitochondria after 24 h of incubation. For distinguishing between endogenous and exogenous mitochondria in BCC experiments, MCF10A were pre-transfected with Mito7–mRuby and BCCs were pre-transfected with COX8–GFP all following Lipofectamine 3000 transfection protocol (L3000001 Thermo Fisher Scientific). Confocal fluorescence images were exported from ImageJ, where all images were processed using a consistent brightness and contrast adjustment. Specifically, the intensity range was adjusted from 0–256 to 0–50 for all images to enhance visualization while maintaining relative contrast. This adjustment was uniformly applied across all images to ensure consistency and prevent bias in image interpretation. No differential scaling was applied between images.

### Colocalization analysis

Colocalization analysis was conducted using the Coloc2 plugin in Fiji/ImageJ (version 1.54f). For each condition (12 h and 24 h), 10 images per biological replicate (*n*=3) were analyzed. Pearson's correlation coefficient, Manders' coefficients, and the Costes significance test values were calculated using Coloc2 with default parameters unless otherwise specified. The Costes threshold and randomization-based significance (200 iterations) were obtained directly from the Coloc2 Costes implementation.

### FLIM acquisition

NADH FLIM images were imaged on Zeiss LSM 880 coupled with a Ti:Sapphire laser for two-photon excitation set at 740 nm wavelength and 80 MHz pulse repetition. A dichroic filter at 690 nm was used to separate fluorescence signal from the laser and emission signal was detected in two channels using a bandpass filter cube 460/80 nm (NADH) and 540/50 nm (FAD). Fluorescence lifetime decays were collected by an A320 FastFLIM box. Images are 256×256 in size and pixel dwell time of 16.38 μs collecting 15 frames per image. Laser scanned lifetime images were all calibrated before and after experiments using Coumarine 6 at 100 μM giving a stable lifetime of τ=2.5 ns. A FLIM box and confocal laser scanning microscopes were synchronized to the same pixel dwell time at 16.53 μs. For each pixel on the image (*i,j*), a photon count histogram is built from the arrival time of the emitted light to generate lifetime decay of that pixel, which is known as time-correlated single-photon counting (TCSPC). The phasor approach Fourier transforms these decay curves into another polar system where (*g,s*) coordinates are determined:

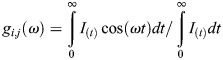


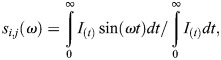
where ω is the pulse repetition of two photon laser excitation at 80 MHz. Raw data were then transformed into R64 files after referencing with Coumarine 6 files which were previously measured at 2.5 ns lifetime on the phasor plot. The phasor plot (*g,s*) coordinates can further derive the relative modulation (*M*) and phase shift (ϕ) arrays of the emission signal from the equation below:

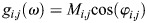


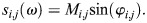
The phasor approach to FLIM analysis was done for whole-cell masking to acquire the single-cell phasor lifetime on the SimFCS software (Laboratory for Fluorescence Dynamics, University of California, Irvine, USA). To acquire single mitochondria lifetime data, we used FIBIS analysis, which enables mitochondria masking, correction for blurring due to movements and the reconfiguring of intensity histograms to account for relatively high intensity pixels.

### Inhibition studies

Glycolytic inhibition was undertaken with 2-deoxyglucose (D8375 Sigma-Aldrich, USA) with a working concentration of 10 mM and set as positive control to bound NADH. OXPHOS inhibition was undertaken with rotenone (AC132370050 Thermo Fisher Scientific), oligomycin (AAJ61898MA Thermo Fisher Scientific, USA) and antimycin A (AAJ63522MA, Thermo Fisher Scientific) with working concentrations of 5 μM, 10 μM and 10 μM, respectively. Rotenone was chosen for the negative control for bound NADH. Each inhibitor was induced and incubated overnight before NADH FLIM imaging.

### Seahorse assay measurement

Cell metabolism quantification was done by measuring respiration and mitochondrial function, such as the OCR and ECAR on an XF Extracellular Flux Analyzer (Agilent, USA). Sample cells were seeded at 10,000 to 12,000 densities in a 24-well Seahorse XF-242 assay plate and changed from culture medium to Seahorse assay medium 1 h before measurement. Cartridge probes were calibrated in a non-CO_2_ incubator for one night with wells filled with 200 μl of XF Calibrant medium before measurement for basal level establishment. OCRs (pMoles/min) were measured during 4 min. The maximum respiration rate was determined by measuring the extracellular OCR after the addition of the uncoupler FCCP. This reflects the highest achievable electron transport and substrate oxidation activity in the cells. The glycolytic reserve was determined by measuring the difference between the ECAR following glucose addition and ECAR after inhibition of mitochondrial ATP synthase using oligomycin. This measurement reflects the metabolic phenotype of the cells and their capacity to transition from mitochondrial respiration to glycolysis in response to ATP demand.

### Cell viability measurements

Cell viability was measured using the XTT Assay (30007 Biotium, USA), which measures the orange absorbance from active mitochondria enzymes. In brief, BCCs were cultured at 4×10^4^ density in 96-well plates and co-cultured with freshly isolated mitochondria for 24 h. Samples were later treated with doxorubicin (D1515-10MG, Sigma-Aldrich) at 1, 2 and 5 μM for 24 h. The XTT Assay medium was made according to the protocol by mixing 25 μl Activation Reagent with 5 ml XTT Solution. Samples were incubated under biological conditions for 2 h before imaging in a plate reader (Biotek Synergy). Absorbance was measured ranging from 450–500 nm, and the subtracted background signal at 600–630 nm. Curve fit was performed using fitting analysis tool from Origin 2023 and setting parameters to dose response mode at log(inhibitor) versus response – variable slope (four parameters).

### TMRM and CellROX analysis

In total, 4×10^4^ cells were seeded on glass bottom eight-well plate (Cellvis, USA). 100 nM TMRM (T668 Thermo Fisher Scientific) was stained in BCC cultures and incubated for 30 min. 500 nM CellROX Deep Red (C10422 Thermo Fisher Scientific) was stained in BCC cultures and incubated for 30 min. Imaging medium was changed to Phenol Red-Free DMEM with 1% heat-inactivated fetal bovine serum before imaging. All fluorescence images were thresholded set at the range 100–65535 using Otsu method in ImageJ for individual mitochondria TMRM and CellROX level analysis. More than 30 cells/replicate were analyzed for each of three biological replicates.

### Statistical analysis

Comparison of OCR, ECAR cellular respiration, and cell viability were done using a two-sample paired one-tailed *t*-test with Excel version 16.51. A two-sample Kolmogorov–Smirnov test was applied to measure significance for individual mitochondria analysis, such as TMRM, CellROX, and the free and bound fraction of NADH. Graphs and other statistical analysis were done using Origin 2022.

### Code availability and AI software usage

The FIBIS program was written in MATLAB (Mathworks, R2021a). The MATLAB GUI FIBIS app and related source code are publicly available on GitHub at https://github.com/KenHuang0820/FIBIS. We acknowledge the use of Zot GPT AI for grammar corrections of the manuscript. After using these services, the authors reviewed and edited the content as needed and take full responsibility for the content of the publication.

## Supplementary Material

10.1242/joces.263702_sup1 Supplementary information
