# Supplementary material for: FLIM intensity-based image segmentation reveals upregulated energy metabolism and chemotherapy sensitivity in MCF-7 cells
Source: J Cell Sci. 2025 Dec 8;138(23):jcs263702. doi: 10.1242/jcs.263702 (PMC12752511; doi:10.1242/jcs.263702)
Supplement: Supplementary information [file joces-138-263702-s1.pdf]

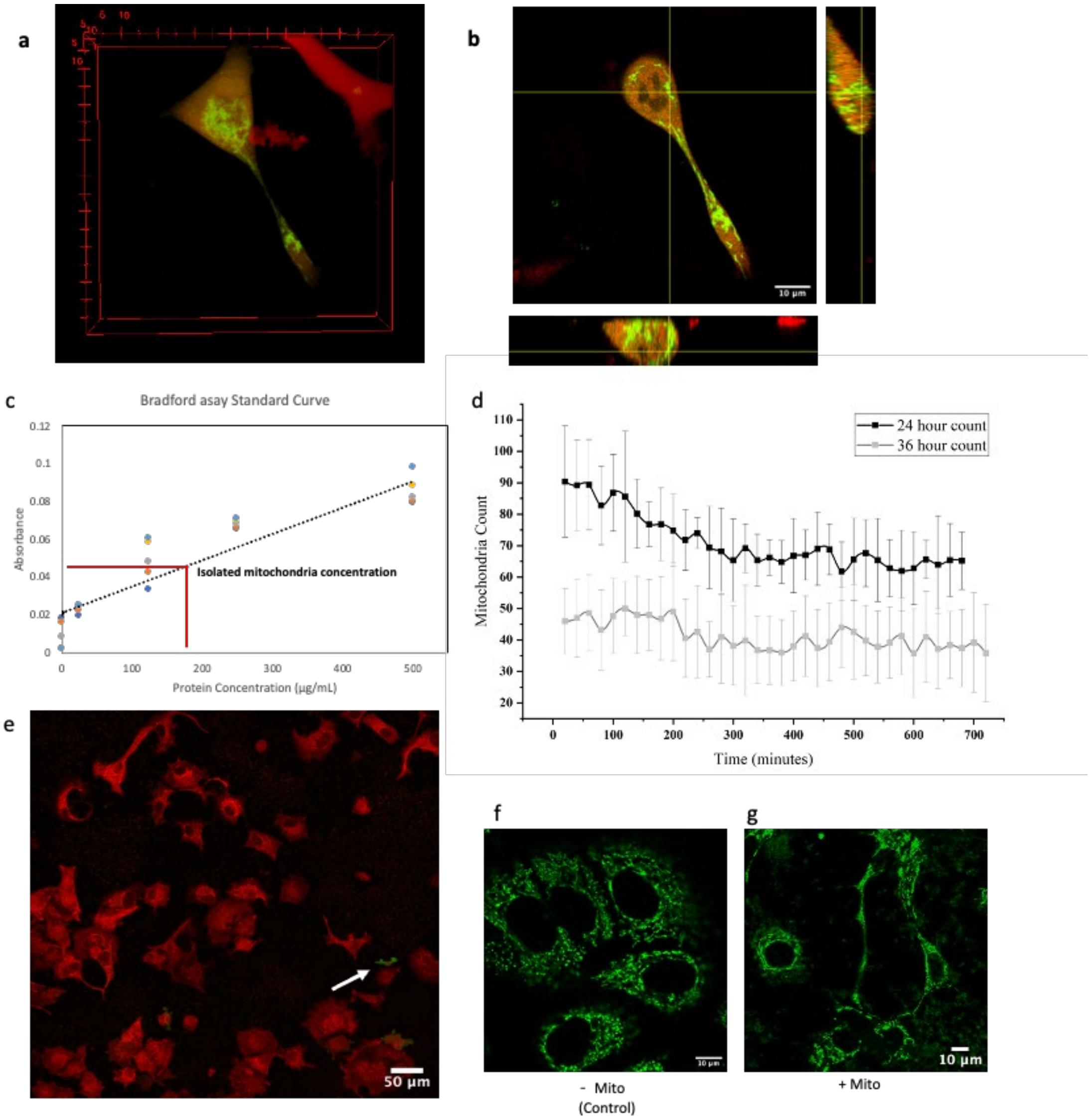

Fig. S1. Fluorescence image of transferred mitochondria in breast cancer cell. a) 3D viewer of pre-labelled epithelial mitochondria with COX8GFP inside of MDA-MB-231 stably expressing mCherry. b) Orthogonal view confirming COX8GFP signal was covered inside of cell body. c) Bradford assay standard curve referenced by BSA with mitochondria protein absorbance at ~0.045. d) Individual mitochondria count according to incubation time. N = 5 location timelapse videos. e) 630x630 µm tile image of MCF7 stained with MitoTracker DeepRed mixed with small portion of Paxilin-GFP from epithelial donor cells. Confocal fluorescence image of f) MCF7 cell culture and g) added with epithelial mitochondria stained with MitoTracker Green where sample group with additional mitochondria shows protruded membrane structure.

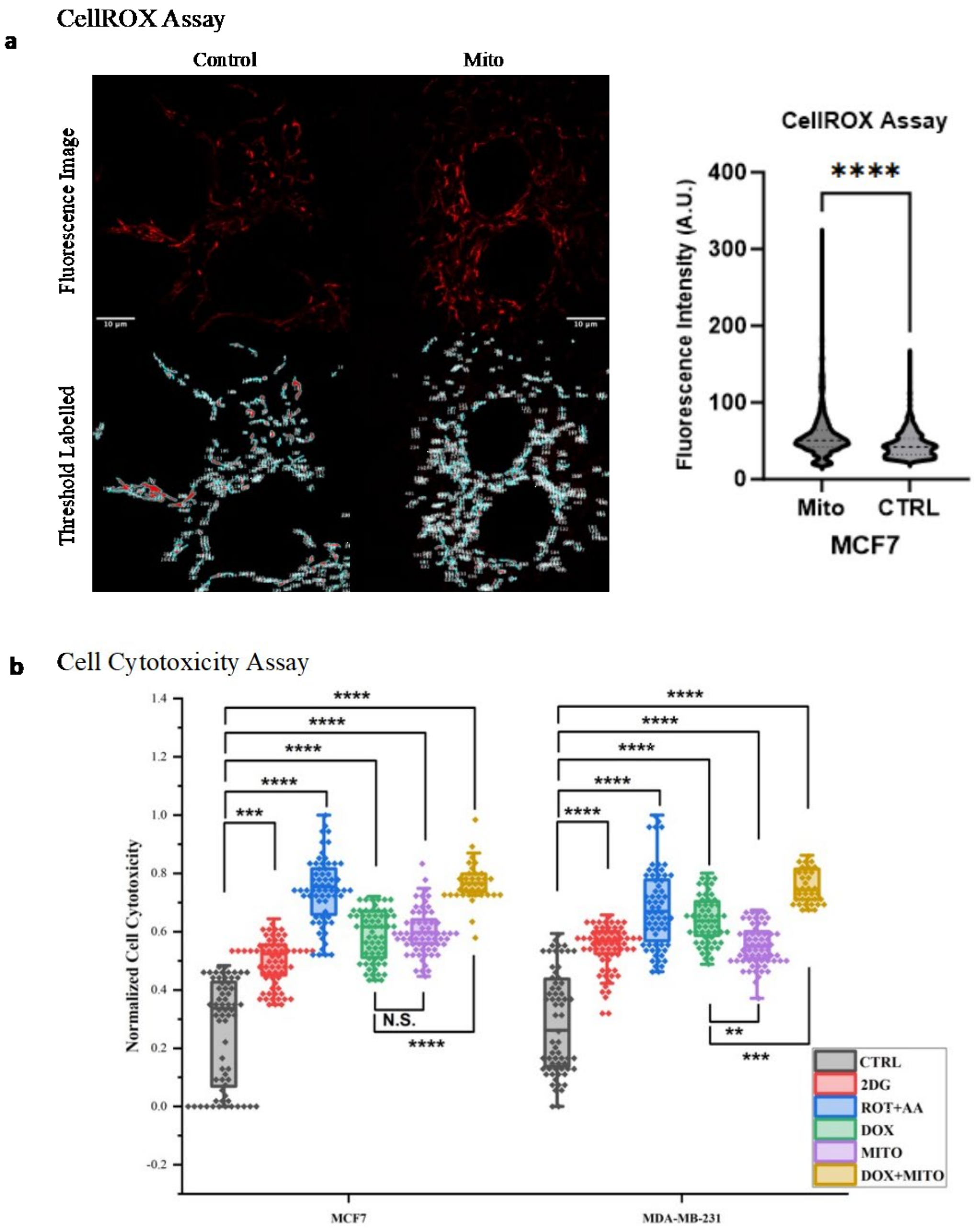

Fig. S2. CellROX and Cell Cytotoxicity assay. a) Single mitochondrial CellROX intensity analysis reveals significant increase in MCF7 Mito group indicating higher levels of reactive oxygen species. N = 2901 and 3087 for MCF7 mitochondria and control group respectively. Data are represented as mean violin plots. p-values are calculated using two sample Kolmogorov-Smirnov test. **** indicates $p < 0.00001$. b) Normalized cell cytotoxicity levels of BCCs treated with metabolic inhibitors (2-deoxyglucose and Rotenone + Antimycin A) and Doxorubicin. Data are represented as mean ± interquartile ranges. Significant p-values were determined by paired two-sample t-test. N = 65 for 3 replicates (** $p < 0.001$, **** $p < 0.00001$).

Journal of Cell Science • Supplementary information

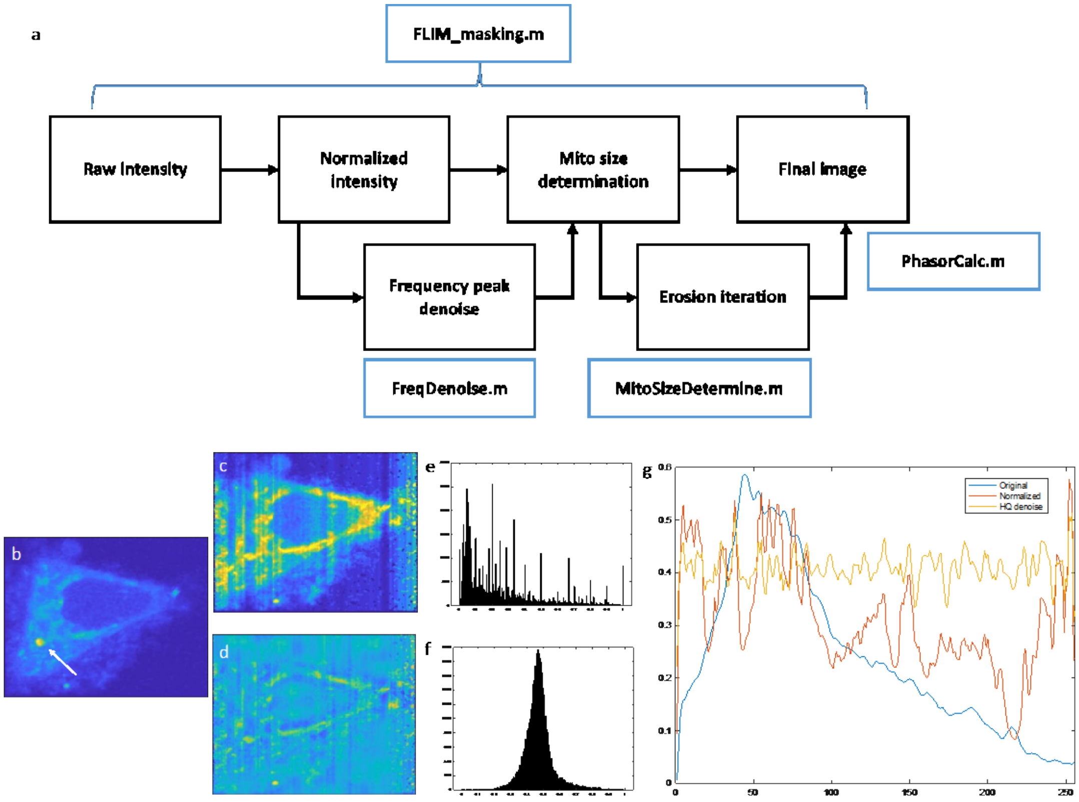

Fig. S3. Flowchart and MATLAB program structure of FIBIS. a) FLIM_masking makes the body of the whole program importing FLIM R64 files, reference read out the image content arrays, and calling of functions. FreqDenoise is called once irregular peaks are detected to smooth histogram and average column intensity across image. MitoSizeDetermine is called when objects area exceeds of 0.5-7μm range to prevent miscalculation of actual mitochondria and erase motion blur. PhasorCalc is called to integrate final segmented image with G arrays for G value analysis. b)Raw intensity image of MCF7 cell where arrow pointed is the area of unexpected high intensityarea decreasing other NADH signatures of the cell. c) Range normalized image which highlights whole frames NADH signature also containing of high frequency noise detected in local peaks of histogram e). Denoise filtered image exhibiting normal distributed histogram for thresholding f). g) Column averaged intensity of the 3 intensity images. Original curve showed the largest peak around the high intensity area. Normalized curve showed frequency noise across normalized image. HQ denoise has smoothed intensity but preserves NADH signature.

Journal of Cell Science • Supplementary information

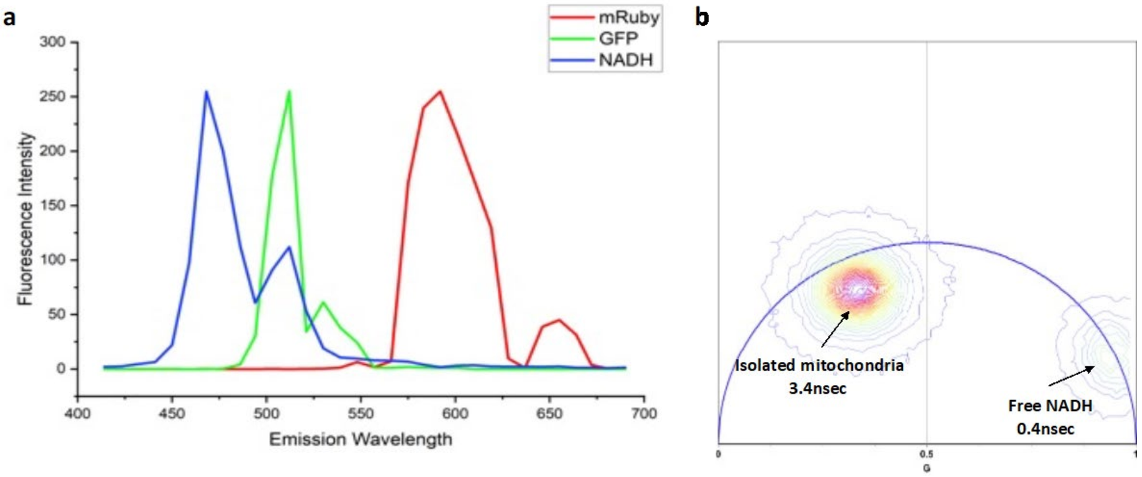

Fig. S4. Emission spectrum of fluorescent probes and Phasor plot calibration of free to bound NADH trajectory. a) Fluorescent probes were excited at 740, 488, and 561 nm wavelength in live cells to measure NADH, GFP, and mRuby emission spectrum respectively using hyperspectral imaging. b) Pure NADH FLIM was measured to locate at 0.4nsec. Isolated mitochondria FLIM was measured within 1 hour after artificial isolation which locates ~3.4nsec.

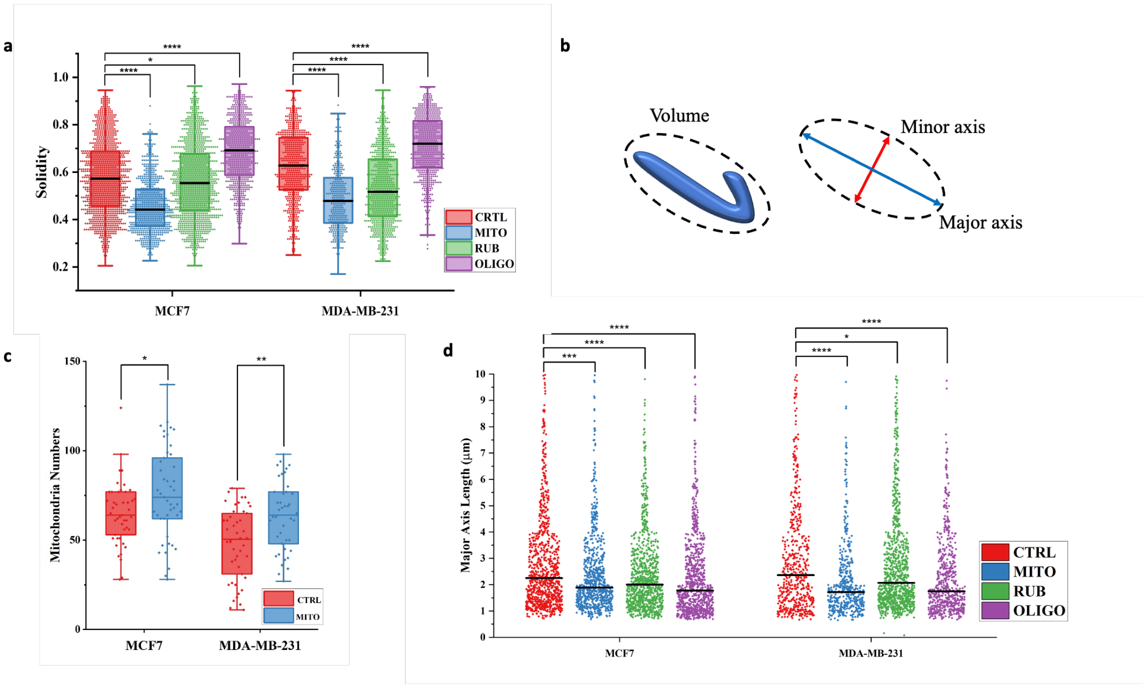

Fig. S5. MitoMeter analysis of mitochondrial dynamics. a) Solidity of BCCs treated with epithelial mitochondria, mitophagy activator Ruxolitinib, and fission activator Oligomycin respectively. Data are represented as mean ± interquartile ranges. p-values are calculated using two sample Kolmogorov-Smirnov test. * indicates $p < 0.05$ and **** indicates $p < 0.00001$. b) Solidity is represented by the ratio of true object pixels to the convex hull (volume) which indicates the branching of mitochondria network. Object and track numbers represent individual mitochondria numbers. Mitochondrial length is calculated by the ratio of major and minor axis length. c) Mitochondrial numbers before and after transferred mitochondria post 24 hours incubation. Data are represented as mean ± interquartile ranges. p-values are calculated using pair sample t test. * indicates p < 0.05 and ** indicates $p < 0.001$. d,e) Mitochondrial length of BCCs treated with epithelial mitochondria, mitophagy activator Ruxolitinib, and fission activator Oligomycin respectively.

Journal of Cell Science • Supplementary information

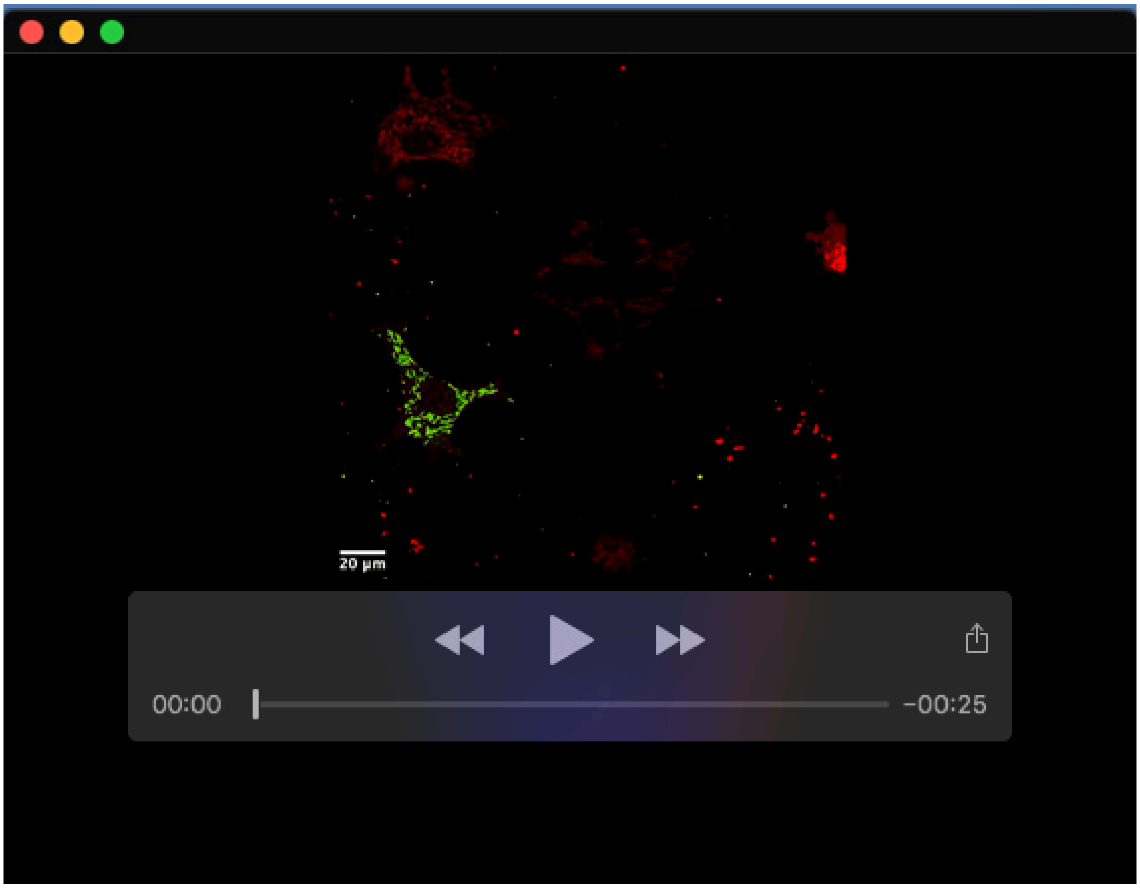

Movie 1. Supporting timelapse video of exogenous mitochondria transfer into BCCs. MCF10A cells were pre-transfected with COX8-GFP, marking existing epithelial mitochondria with green fluorescence. BCCs were pre-transfected with Mito7-mRuby, marking their mitochondria with red fluorescence. The timelapse video, taken from 36 to 48 hours post mitochondria transfer at 20-minute intervals, shows the interaction and integration of exogenous mitochondria into BCCs.

Journal of Cell Science • Supplementary information
